# The anemia-independent impact of myelodysplastic syndromes on health-related quality of life

**DOI:** 10.1007/s00277-021-04654-1

**Published:** 2021-09-02

**Authors:** Hanneke J. C. M. Wouters, Annette Conrads-Frank, Karin A. Koinig, Alex Smith, Ge Yu, Theo de Witte, Bruce H. R. Wolffenbuttel, Gerwin Huls, Uwe Siebert, Reinhard Stauder, Melanie M. van der Klauw, on behalf of the MDS-RIGHT partners

**Affiliations:** 1 Department of Hematology, University of Groningen, University Medical Center Groningen, Groningen, The Netherlands; 2 Department of Endocrinology, University of Groningen, University Medical Center Groningen, 9700 RB Groningen, The Netherlands; 3 Institute of Public Health, Medical Decision Making and Health Technology Assessment, Department of Public Health, Health Services Research and Health Technology Assessment, UMIT - University for Health Sciences, Medical Informatics and Technology, Hall in Tirol, Austria; 4 Department of Internal Medicine V (Hematology and Oncology), Medical University Innsbruck, Innsbruck, Austria; 5 Epidemiology and Cancer Statistics Group, Department of Health Sciences, University of York, York, UK; 6 Population Health Sciences Institute, Newcastle University, Newcastle upon Tyne, UK; 7 Department of Tumor Immunology, Radboud University Medical Center, Nijmegen, The Netherlands; 8 Division of Health Technology Assessment, ONCOTYROL - Center for Personalized Cancer Medicine, Innsbruck, Austria; 9 Center for Health Decision Science, Department of Health Policy and Management, Harvard T.H. Chan School of Public Health, Boston, MA USA; 10 Institute for Technology Assessment and Department of Radiology and Department of Radiology, Massachusetts General Hospital, Harvard Medical School, Boston, MA USA

**Keywords:** Myelodysplastic syndromes, Health-related quality of life, Anemia, Hemoglobin

## Abstract

Myelodysplastic syndromes (MDS) are in the majority of cases characterized by anemia. Both anemia and MDS per se may directly contribute to impairments in health-related quality of life (HRQoL). In this study, we aimed to investigate the anemia-independent impact of MDS on HRQoL. We evaluated participants (≥ 50 years) from the large population-based Lifelines cohort (*N* = 44,694, mean age 59.0 ± 7.4 years, 43.6% male) and the European MDS Registry (EUMDS) (*N* = 1538, mean age 73.4 ± 9.0 years, 63.0% male), which comprises a cohort of lower-risk MDS patients. To enable comparison concerning HRQoL, SF-36 scores measured in Lifelines were converted to EQ-5D-3L index (range 0–1) and dimension scores. Lower-risk MDS patients had significantly lower HRQoL than those from the Lifelines cohort, as illustrated in both the index score and in the five different dimensions. Multivariable linear regression analysis demonstrated that MDS had an adjusted total impact on the EQ-5D index score (B =  − 0.12, *p* < 0.001) and an anemia-independent “direct” impact (B =  − 0.10, *p* < 0.001). Multivariable logistic regression analysis revealed an anemia-independent impact of MDS in the dimension mobility, self-care, usual activities, and anxiety/depression (all except pain/discomfort). This study demonstrates that the major part of the negative impact of lower-risk MDS on HRQoL is not mediated via anemia. Thus, the therapeutic focus should include treatment strategies directed at underlying pathogenic mechanisms to improve HRQoL, rather than aiming predominantly at increasing hemoglobin levels.

**Supplementary Information:**

The online version contains supplementary material available at 10.1007/s00277-021-04654-1.

## Introduction

Myelodysplastic syndromes (MDS) are acquired clonal disorders of the hematopoietic stem cell, which are characterized by bone marrow failure resulting in blood cytopenias. Anemia is the most prevalent cytopenia and is the most common objective disease manifestation [1, 2]. Several studies have been conducted to assess health-related quality of life (HRQoL) in patients with MDS. HRQoL is usually decreased in patients with MDS and is associated with an unfavorable survival [3, 4]. A previous study comparing HRQoL in newly diagnosed individuals with lower-risk MDS (LR-MDS) (*N* = 1690) demonstrated that a significant proportion of MDS patients experience profound age- and sex-dependent restrictions in different HRQoL dimensions [5].

Patients with MDS are classified into subgroups based on several biological parameters to predict overall survival and the risk of transformation to acute myeloid leukemia (Revised International Prognostics Scoring System; IPSS-R). Especially individuals with higher IPSS-R risk MDS have a poor prognosis [6, 7]. Intervention goals differ between IPSS-R risk groups. Treatment for patients with LR-MDS is focused on restoration of cytopenias, to delay progression to acute myeloid leukemia and prolonging survival [8]. The only curative option is hematopoietic cell transplantation [1]. Due to advanced age and/or co-existing medical conditions, patients are often ineligible for this procedure. Therefore, treatment decisions for lower risk IPSS-R MDS often focus on improvement of quality of life (QoL) [9].

Anemia, especially anemia of chronic inflammation, has been associated with a decreased HRQoL in older individuals in the general population [10]. In MDS patients, both anemia and other anemia-independent characteristics of MDS may have a direct contribution to impairments in HRQoL. There is conflicting evidence on the impact of hemoglobin levels on QoL in MDS, whereas chronic RBC transfusion dependency is clearly associated with an inferior QoL [11]. Steensma et al. demonstrated a poor correlation between hemoglobin levels and fatigue in MDS patients (*N* = 359). However, the influence of prior RBC transfusions on the evaluated hemoglobin levels was uncertain [12]. In another study (*N* = 280), the degree of anemia was associated with, but could not fully explain, the subjective perception of fatigue [13]. In smaller studies (*N* = 50 and *N* = 39), lower hemoglobin levels were associated with fatigue and poorer QoL [14, 15]. Further, intervention studies do not convincingly prove that interventions that aim to increase the hemoglobin levels in MDS patients have a beneficial effect on HRQoL [16, 17].

Since hemoglobin levels may not be the main determinant of HRQoL in MDS patients, studies assessing the impact of other mechanisms are warranted [18]. In this study, we aimed to investigate the anemia-independent impact of LR-MDS on HRQoL, using the European MDS (EUMDS) Registry, and the Lifelines cohort.

## Methods

### Study design

First, we analyzed data from the EUMDS Registry, a non-interventional longitudinal study, enrolling newly diagnosed patients with MDS (IPSS low or intermediate-1) from 148 hematology centers in 16 European countries and Israel (started in 2007). Enrollment was within 100 days of the diagnostic bone marrow aspirate. The study has been approved by the local ethics committees and written informed consent was obtained from all participants. The study has been performed in accordance with the Declaration of Helsinki. Details of this cohort and the recorded clinical parameters have been described elsewhere [4, 5]. For the current analysis, we used data from the baseline survey and excluded participants younger than 50 years and those with missing data on HRQoL or hemoglobin level. Individuals who received a RBC transfusion within 30 days before the blood sample was taken were also excluded. In total, 1538 EUMDS patients were eligible for analysis. Second, we analyzed data from the Lifelines cohort. Lifelines is a multi-disciplinary prospective population-based cohort study examining in a unique three-generation design the health and health-related behaviors of 167,729 persons living in the North of The Netherlands. It employs a broad range of investigative procedures in assessing the biomedical, socio-demographic, behavioral, physical, and psychological factors which contribute to the health and disease of the general population, with a special focus on multi-morbidity and complex genetics. The local ethics committee approved the research protocol, and informed consent was signed by every participant [19, 20]. At baseline, all subjects completed a self-administered questionnaire, amongst others on medical history, past and current diseases, use of medication, and quality of life. For the present study, we included 44,694 subjects of ≥ 50 years from whom data on HRQoL and hemoglobin level were available. Data about red blood cell transfusions were not available for Lifelines participants.

### Laboratory parameters

In Lifelines, blood samples were collected in the morning after an overnight fast. The blood samples were placed at 4 °C and transported from the Lifelines research site to the Lifelines laboratory, under tightly controlled and continuously monitored conditions. From the Lifelines laboratory, part of the samples were directly transferred to the central laboratory of the University Medical Center Groningen, to perform routine clinical chemistry assays on fresh samples. Hemoglobin, total leucocytes, and thrombocytes were measured using routine procedures on a XE2100-system (Sysmex, Japan). In EUMDS, a complete blood count was performed at entry of the study as part of routine clinical care in the local hospital.

### Scoring of comorbidities

In the EUMDS Registry, comorbidity was scored using the Hematopoietic Cell Transplantation-Comorbidity Index (HCT-CI) and the Myelodysplastic Syndrome-Comorbidity Index (MDS-CI). The HCT-CI was developed by Sorror et al. and comprises 17 groups of diseases [21, 22]. The MDS-CI was created using the five significant HCT-CI disease groups associated with non-leukemic deaths (cardiac disease (2 points), hepatic disease (1 point), pulmonary disease (1 point), renal disease (1 point), and solid tumor (1 point)) in a population of MDS patients. The final scores are low (0 points), intermediate (1 or 2 points), or high (3 or more points) [23]. Comorbidity index scores were generated based on information given by physicians in the EUMDS registry, and based on self-report combined with verified medication use (scored by ATC code) and laboratory data in the Lifelines cohort. Participants in the Lifelines cohort may suffer from a wide range of medical issues since only individuals with a limited life expectancy or severe psychiatric/physical illness were excluded from participation [24]. The used definitions are listed in Table S1. Complete data were available for 15,784 Lifelines and 1157 EUMDS participants. Demographic characteristics between individuals with and without information available to generate the MDS-CI did not relevantly differ in both cohorts.

### Health-related quality of life

In EUMDS, HRQoL was measured by the EuroQol Five-Dimensional Questionnaire (EQ-5D-3L). The EQ-5D-3L is a commonly used, generic HRQoL questionnaire consisting of a descriptive part and a visual analogue scale (VAS). The descriptive part consists of five dimensions related to daily activities (mobility, self-care, usual activities, pain/discomfort, and anxiety/depression), with three response categories (no problems, moderate problems, or severe problems) [25]. These questions provide 243 possible health states from which an index score (generally ranging from 0–1) can be calculated [26]. The current analyses were performed using the UK value set [27]. In Lifelines, HRQoL was measured using the RAND 36-Item Health Survey (Dutch version of the Short-Form 36 (SF-36) questionnaire). This generic questionnaire consists of 36 items in eight dimensions: physical functioning (10 items), physical role functioning (4 items), bodily pain (2 items), general health (5 items), vitality (4 items), social functioning (2 items), emotional role functioning (3 items), and mental health (5 items). Dimension scores (range 0–100) on the eight domains were generated [28]. A higher score reflects a better health status and well-being in both questionnaires. The SF-12 is a shortened version of the SF-36, including 12 of the 36 questions [29].

### Conversion of SF-36 to EQ-5D

To enable comparisons between HRQoL measured in EUMDS and in Lifelines, the SF-36 data (and derived SF-12 data) were converted to EQ-5D data in two approaches. In the first approach, domain scores of the SF-36 questionnaire were converted into a preference-based EQ-5D index score following the mapping algorithm of Rowen et al. (random effects generalized least squares model 3) [30]. In the second approach, the multinomial response mapping algorithm developed by Gray et al. was used to estimate the probability of a score within one of the three response categories of the EQ-5D for each dimension based on the SF-12 results [31].

### Statistical analysis

**Fig. 1:**
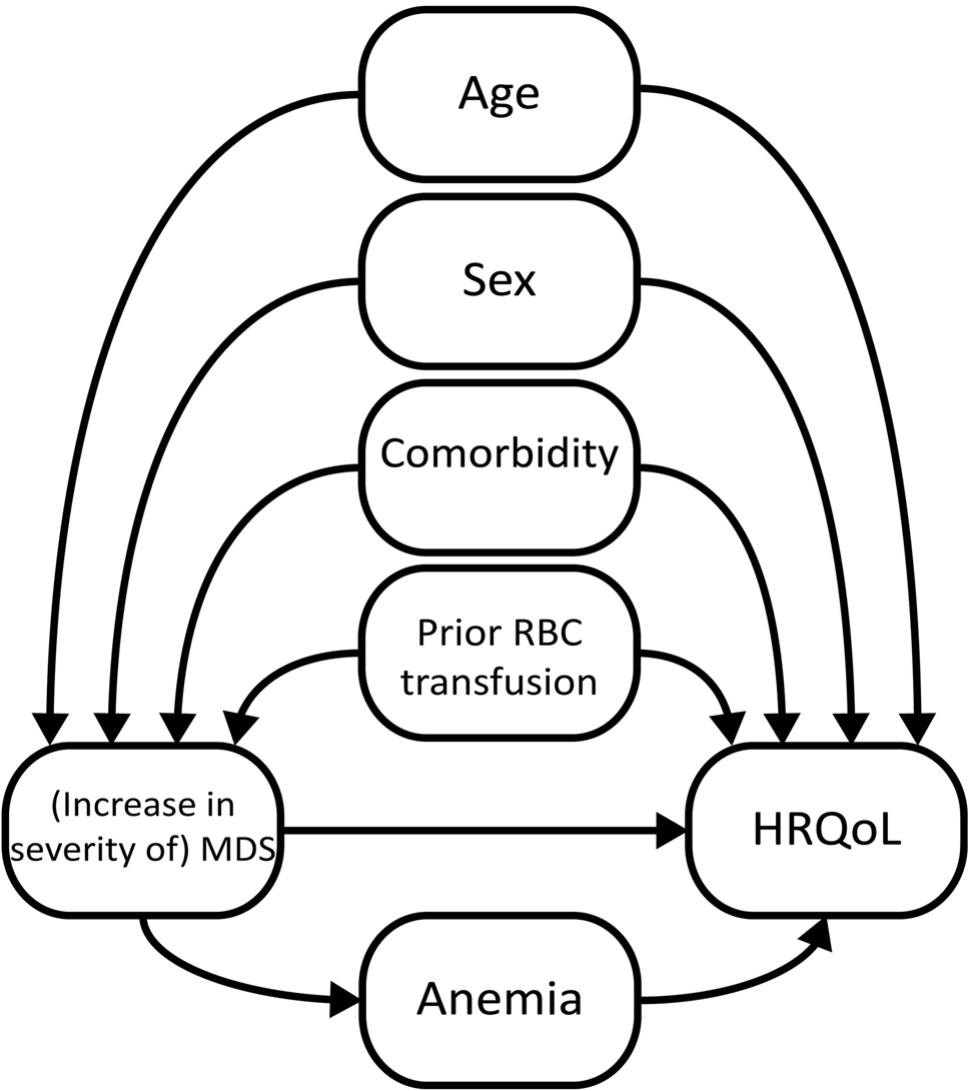
Directed acyclic graph for the assessment of the total and anemia-independent impact of lower-risk myelodysplastic syndromes on health-related quality of life. This causal diagram encodes the prior assumptions on common causes of (increase in severity of) MDS and HRQoL as well as direct and indirect causal pathways from MDS to HRQoL. The four confounders may also influence anemia; the related arrows have been left out for simplified visualization but are fully considered in the analyses. HRQoL, health-related quality of life; MDS, myelodysplastic syndromes; RBC, red blood cell

Data were presented as mean (standard deviation), median (interquartile range), or percentage. We evaluated between-group differences using one-way ANOVA, Kruskal–Wallis test, or Chi-square test, as appropriate. Anemia was defined according to the WHO criteria as a hemoglobin level < 13.0 g/dL in men and < 12.0 g/dL in women [32]. Following best practice recommendations for observational studies [33], a simple causal diagram was derived by an expert panel of epidemiologists and clinical experts (authors) to explicitly present the prior assumptions based on literature in order to derive the causal effect of MDS on HRQoL [5]. The causal directed acyclic graph (DAG) for the study is presented in Fig. 1. Based on the causal DAG, confounders (i.e., common causes of (severity of) MDS and HRQoL) were selected prior to the analysis. Multivariable linear regression analyses (for the outcome EQ-5D index score) and logistic regression analyses (for the outcomes EQ-5D domains; problems (moderate or severe) versus no problems) were used to examine both the total and the anemia-independent impact of MDS on HRQoL. First, a crude (unadjusted) analysis for the association between MDS and HRQoL was performed (model 1). Model 2 was adjusted for the confounders age (50–60/61–75/ > 75 years), sex, comorbidity (MDS-CI low/intermediate/high), and prior RBC transfusions, in order to assess the total causal impact of MDS on HRQoL. In model 3, we additionally controlled for the mediator anemia to assess the (“direct”) causal impact of MDS on HRQoL that is not mediated via anemia. Lastly, in model 4 we added hemoglobin levels to the model. Participants were eligible for inclusion in the EUMDS registry when they had a low or intermediate‐1 risk score according to the IPSS. From 2012, IPSS‐R scores were retrospectively assigned by one of the investigators of the EUMDS registry and verified by an independent expert of the international IPSS working group of the MDS Foundation [4]. As sensitivity analysis, the analyses were repeated excluding individuals who were retrospectively classified as IPSS-R intermediate, high or very high risk (*N* = 205), or who had an unknown IPSS-R (*N* = 142), and individuals who ever received a RBC transfusion (*N* = 313). Unstandardized regression coefficients and odds ratios are reported with 95% confidence intervals. All statistical tests were performed two-sided and a *p*-value < 0.05 was considered significant. Data were analyzed using IBM SPSS software, version 23.0.

## Results

Relevant baseline characteristics for both anemic and non-anemic participants from the population-based Lifelines cohort and EUMDS cohort are shown in Table 1. Participants from the EUMDS cohort were significantly older, were more often male, and revealed a higher comorbidity burden compared with the Lifelines participants. Anemia was present in 89.2% of EUMDS participants in contrast to 2.9% in individuals of Lifelines. In both cohorts, anemic participants were older than non-anemic individuals, whereas in the Lifelines cohort, there was a higher percentage of anemic females. Anemic EUMDS participants had significantly lower mean hemoglobin levels than anemic Lifelines participants.

**Table 1:** Baseline characteristics of the Lifelines cohort and the EUMDS cohort

	**Lifelines participants**				**EUMDS participants**				**Lifelines vs. EUMDS**
	**Total cohort**	**Anemic individuals (*N* = 1294)**	**Non-anemic individuals (*N* = 43,400)**	***P *value**	**Total cohort**	**Anemic individuals (*N* = 1372)**	**Non-anemic individuals (*N* = 166)**	***P *value**	***P *value**
**Age**				< 0.001				< 0.001	< 0.001
50–60 years (%)	56.0	57.0	56.0		9.3	8.7	14.5		
61–75 years (%)	41.5	37.3	41.6		45.3	44.0	56.0		
> 75 years (%)	2.5	5.7	2.4		45.4	47.3	29.5		
**Male (%)**	43.6	26.9	44.1	< 0.001	63.0	63.0	62.7	0.92	< 0.001
**Hemoglobin level (g/dL)**									
Male	15.0 ± 1.0	12.2 ± 0.7	15.1 ± 0.9	< 0.001	10.6 ± 1.9	10.2 ± 1.5	14.0 ± 0.9	< 0.001	< 0.001
Female	13.6 ± 0.9	11.3 ± 0.8	13.7 ± 0.8	< 0.001	10.1 ± 1.6	9.8 ± 1.2	13.0 ± 0.7	< 0.001	< 0.001
**RBC transfusion (%)**	NA	NA	NA	NA	20.4	22.6	1.8	< 0.001	NA
**MDS-CI**^**a**^** (%)**				0.21				0.39	< 0.001
Low	78.1	75.0	78.2		61.7	61.1	66.7		
Intermediate	20.2	23.3	20.1		32.9	33.3	30.0		
High	1.7	1.7	1.7		5.4	5.6	3.3		

Data are given as mean ± SD or percentage. ^a^MDS-CI was available in 516 anemic Lifelines participants, in 15,268 non-anemic Lifelines participants, in 1037 anemic EUMDS participants and in 120 non-anemic EUMDS participants. *MDS-CI*, myelodysplastic syndromes comorbidity index; *NA*, not applicable; *RBC*, red blood cell; *SD*, standard deviation

**Fig. 2:**
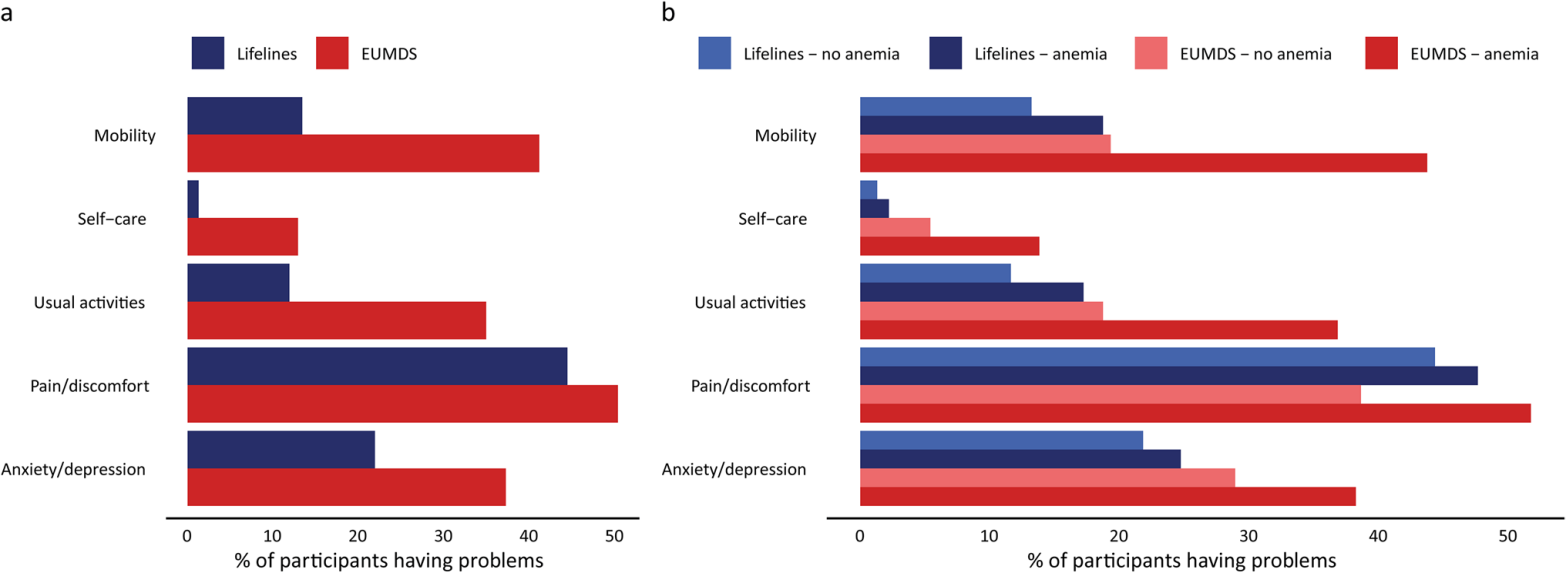
Percentage of individuals with moderate to severe problems in the five EQ-5D dimensions according to cohort and presence of anemia. Panel **a** shows the percentage of individuals with moderate to severe problems in the five EQ-5D dimensions according to cohort (EUMDS or Lifelines). Panel **b** shows the percentage of individuals with moderate to severe problems in the five EQ-5D dimensions according to the presence of anemia in both cohorts

The EUMDS cohort was characterized by a mean EQ-5D index score of 0.74 (± 0.27), whereas the mean EQ-5D index score in the Lifelines cohort was 0.90 (± 0.10) (*P* < 0.001) (Table S2). Likewise, a significantly larger percentage of EUMDS participants reported moderate to severe problems in all five EQ-5D dimensions compared to individuals from the Lifelines cohort (Fig. 2a). The most pronounced differences were observed in the dimensions mobility and usual activities. Smaller differences were observed in the dimensions self-care, pain/discomfort, and anxiety/depression.

A lower EQ-5D index score as well as a larger percentage of individuals with moderate to severe problems was observed in females, at advanced age, and in individuals with an increased comorbidity burden in all five EQ-5D dimensions in both cohorts, except for the dimension anxiety/depression in the EUMDS cohort. In this dimension a larger proportion of problems was only observed in females compared to males and no significant differences related to age or comorbidity burden were present (Table S2), Furthermore, a significantly lower index score and a larger percentage of participants with moderate to severe problems in all five dimensions were detected in anemic participants as compared to non-anemic participants in both cohorts, being more pronounced in the EUMDS cohort (Fig. 2b and Table S2).

To assess the anemia-independent impact of MDS on HRQoL, univariable and multivariable linear and logistic regression analyses were performed. Univariable linear regression analysis demonstrated a significant negative association between MDS and EQ-5D index scores in the crude analysis (model 1, B =  − 0.16). MDS had an adjusted (causal) total impact on HRQoL (model 2, B =  − 0.12) and an anemia-independent “direct” impact (model 3, B =  − 0.11), independent of age, sex, comorbidity, and prior RBC transfusions. Since the hemoglobin levels differed significantly between anemic individuals in both cohorts, in model 4, we additionally adjusted for hemoglobin levels, which did not change the impact as present in model 3 (model 4, B =  − 0.10) (Table 2). Our results indicated that the presence of MDS was associated with a decrease of EQ-5D index score of 0.10 on a range of 0 − 1, independent of anemia.

**Table 2:** The total and anemia-independent “direct” impact of lower-risk myelodysplastic syndromes and the impact of anemia on HRQoL (EQ-5D index score) based on univariable and multivariable linear regression analyses

EQ-5D index score																
	**Model 1 (crude association)**				**Model 2 (total causal impact)**				**Model 3 (“direct” causal impact)**				**Model 4 (“direct” causal impact)**			
	B	SE	β	*P* value	B	SE	β	*P* value	B	SE	β	*P* value	B	SE	β	*P* value
**MDS (y/n)**	− 0.163	0.003	− 0.259	< 0.001	− 0.119	0.004	− 0.243	< 0.001	− 0.106	0.006	− 0.215	< 0.001	− 0.104	0.006	− 0.212	< 0.001
**Anemia (y/n)**									− 0.016	0.005	− 0.038	< 0.001	− 0.013	0.005	− 0.031	0.012
**Hemoglobin level (g/dL)**													0.001	0.001	0.015	0.23
**Age group**																
50–60 years					Ref				Ref				Ref			
61–75 years					0.005	0.002	0.019	0.009	0.005	0.002	0.019	0.009	0.005	0.002	0.019	0.009
> 75 years					0.037	0.005	− 0.066	< 0.001	− 0.036	0.005	− 0.064	< 0.001	− 0.036	0.005	− 0.064	< 0.001
**Sex (female)**					− 0.028	0.002	− 0.112	< 0.001	− 0.028	0.002	− 0.110	< 0.001	− 0.026	0.002	− 0.104	< 0.001
**MDS-CI**																
Low					Ref				Ref				Ref			
Intermediate					− 0.037	0.002	− 0.121	< 0.001	− 0.037	0.002	− 0.120	< 0.001	− 0.037	0.005	− 0.121	< 0.001
High					− 0.089	0.006	− 0.100	< 0.001	− 0.089	0.006	− 0.100	< 0.001	− 0.089	0.006	− 0.100	< 0.001
**RBC transfusion (y/n)**					− 0.077	0.008	− 0.074	< 0.001	− 0.075	0.008	− 0.072	< 0.001	− 0.073	0.009	− 0.070	< 0.001

Model 1 was a crude analysis. Model 2 was adjusted for age (50–60 years, 61–75 years, or 75 years and older), sex, comorbidity (MDS-CI low, intermediate, high), and prior RBC transfusions. Model 3 was additionally adjusted for anemia. Model 4 was additionally adjusted for hemoglobin levels. Anemia was defined as a hemoglobin level < 13.0 g/dL in men and < 12.0 g/dL in women. B, unstandardized regression coefficient; β, standardized regression coefficient; *MDS*, myelodysplastic syndromes; *MDS-CI*, myelodysplastic syndromes comorbidity index; *RBC*, red blood cell; *Ref*, reference; *SE*, standard error

**Fig. 3:**
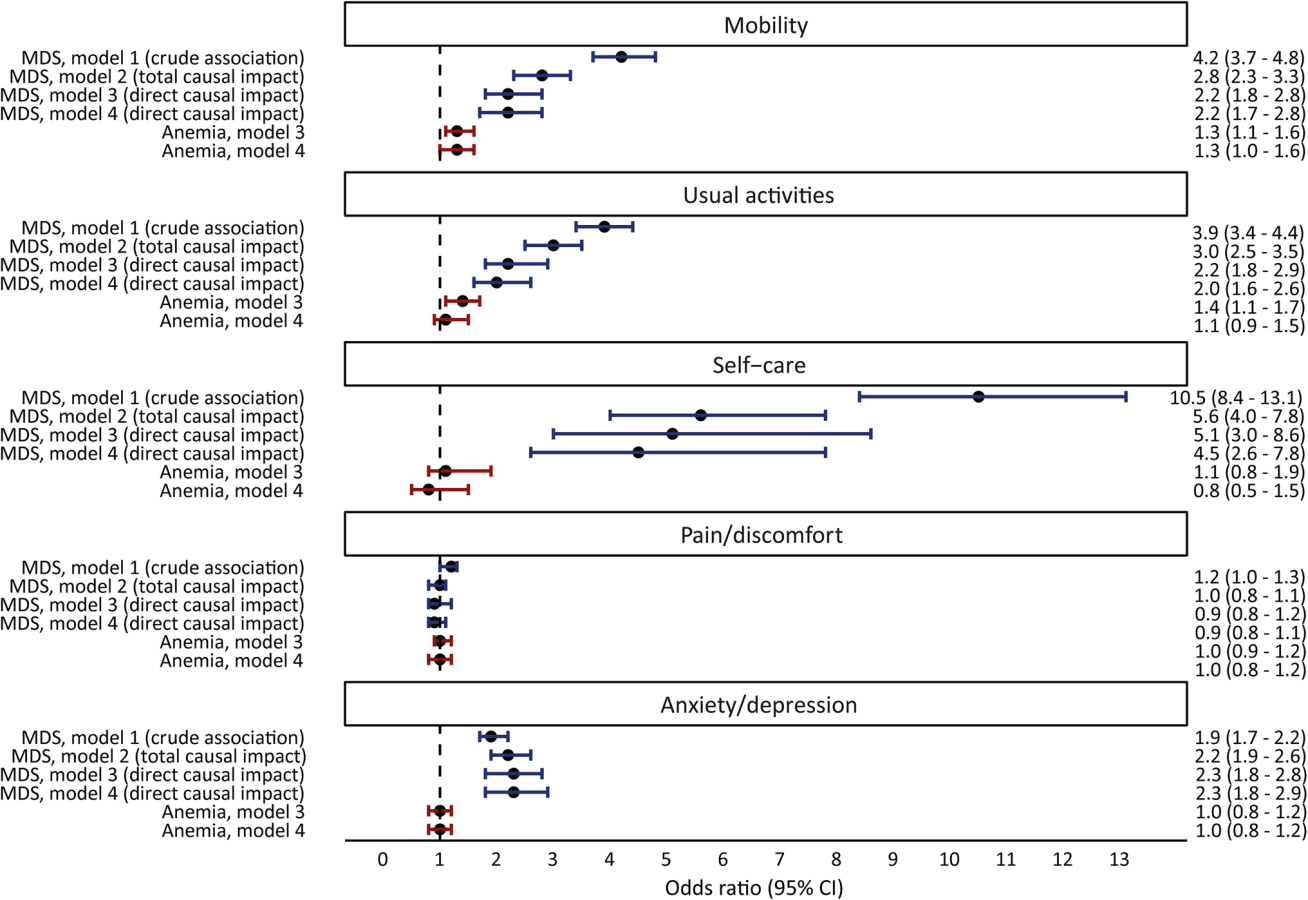
Forest plot showing the total and anemia-independent impact of lower-risk myelodysplastic syndromes and the impact of anemia on health-related quality of life. Forest plots indicating the odds ratio (circles) and 95% confidence intervals (error bars) of having moderate to severe problems in the five dimensions of the EQ-5D as crude association between MDS and HRQoL (blue, model 1), total causal impact of MDS on HRQoL (blue, model 2), anemia-independent “direct” causal impact of MDS (blue, model 3 and 4), and impact of anemia (red, model 3 and 4). Model 1 was an unadjusted (crude) analysis. Model 2 was adjusted for age (50–60 years, 61–75 years, or 75 years and older), sex, comorbidity (MDS-CI low, intermediate, high), and prior red blood cell transfusions. Model 3 was additionally adjusted for anemia. Model 4 was additionally adjusted for hemoglobin levels. CI, confidence interval; MDS, myelodysplastic syndromes

Subsequently, logistic regression analysis was performed to assess the anemia-independent impact on the five EQ-5D dimensions. In the univariable analysis (model 1), assessing the unadjusted association between MDS and HRQoL, MDS was strongly associated with decreased HRQoL in four dimensions (all except pain/discomfort). Multivariable regression revealed a significant total adjusted impact of MDS (model 2) in the dimension mobility, self-care, usual activities, and anxiety/depression. Furthermore, increasing age (> 75 years), being female, having a higher MDS-CI, and prior RBC transfusion negatively impacted HRQoL. However, increasing age was associated with less problems in the dimension anxiety/depression. In model 3, we additionally controlled for the mediator anemia, to assess the “direct” causal impact of MDS on the HRQoL dimensions that is not mediated via anemia. An anemia-independent impact of MDS was demonstrated in the same four dimensions; mobility, self-care, usual activities, and anxiety/depression. Additionally, controlling for hemoglobin levels (model 4) decreased the odds ratio’s in the dimensions self-care and usual activities, whereas the odds ratios in the dimensions mobility and anxiety/depression were comparable. Anemia (model 4) had only a residual significant impact on HRQoL in the dimension mobility (Fig. 3 and Table S3). A higher hemoglobin level (per g/dL) was associated with a lower percentage of participants with moderate to severe problems in the dimensions self-care and usual activities. Sensitivity analysis restricted to individuals with IPSS-R (very) low risk and without RBC transfusions did not materially alter the described associations (Table S4).

## Discussion

Combining data from a large number of newly diagnosed LR-MDS patients and a large general population-based cohort, we confirmed the negative impact of LR-MDS on HRQoL, which was particularly present in the dimension mobility, usual activities, self-care, and anxiety/depression. This study is the first to assess the anemia-independent impact of LR-MDS on HRQoL and demonstrates that the major part of the negative impact is not mediated via anemia. After correction for hemoglobin levels, only the dimension mobility was negatively affected by anemia. Previous studies focused on demonstrating the negative impact of MDS in general on HRQoL, while others assessed the impact of anemia and hemoglobin levels on HRQoL in MDS patients, with conflicting results [5, 12–15].

The anemia-independent negative impact of LR-MDS was mainly present in HRQoL dimensions which reflect capacities that are essential for living independently and maintaining social well-being, whereas there was no impact in the dimension pain/discomfort. Especially, the strong impact in the dimension self-care is clinically important, since problems in this dimension are rare in the general population. The impact shown in these dimensions is in line with the fact that fatigue is the most prevalent symptom of MDS [12, 13, 34] and is known to be significantly related to activities of daily living [35]. Additionally, patients with fatigue experience greater overall symptom burden, particularly loss of appetite, dyspnea, insomnia, and pain [13]. The impact of LR-MDS on anxiety/depression decreased when adjusted for age. This may be explained by the known inverse relationship between emotional distress and age in cancer patients. It has been hypothesized that older individuals might be cognitively and emotionally better prepared to accept illness [36].

The small residual impact of anemia in LR-MDS on HRQoL was present in the dimension mobility, whereas hemoglobin levels were associated with HRQoL dimensions self-care and usual activities. This is in line with the impact of anemia in older individuals from the general population, which is also mainly present in dimensions representing physical functioning [10]. It could be hypothesized that the relative effect of anemia on HRQoL might be smaller due to the frequent co-existing morbidities in older MDS patients. In current guidelines for symptomatic LR-MDS patients with anemia, treatment modalities which aim to increase hemoglobin levels are the first choice [1]. Treatment selection depends on the del(5q) status, the transfusion need, and serum EPO levels. Treatment options include erythropoiesis-stimulating agents, granulocyte-colony-stimulating factor (G-CSF), erythropoiesis-maturating agents (luspatercept for MDS with ring sideroblasts, FDA and EMA approved), or immunomodulatory drugs (for MDS with 5q-), which all may be combined with supportive RBC transfusions. Patient‐reported outcome measures, such as HRQoL, are used to assess the effectiveness of an intervention measured from the patient perspective [37]. However, clinical trials assessing these treatment options are not conclusive. A randomized controlled trial assessing the effect of erythropoietin and G-CFS compared to best supportive care did not show a difference in HRQoL between both treatment groups [16]. In contrast, in a prospective study, responders to erythropoietin in combination with G-CSF were shown to have better HRQoL [38]. On the other hand, no beneficial effect on HRQoL of darbepoetin compared to only supportive care was demonstrated [17]. For asymptomatic LR-MDS patients with anemia, watchful waiting is currently recommended. Data from the current study suggest that concerning HRQoL, regardless of anemia status, future studies are needed to identify underlying pathophysiological mechanisms.

In recent years, accumulating evidence has indicated that dysregulation of the immunological environment has an important role in the pathogenesis of MDS [39]. A recent meta-analysis demonstrated increased levels of TNF‐α, IL-6, and IL-8 and decreased levels of IL-17 in individuals with MDS as compared to controls [40]. Several studies assessed the impact of inflammation on HRQoL/self-rated health in older individuals from the general population, showing in general a negative impact. Those studies included different biochemical markers of inflammation, e.g., pro-inflammatory cytokines (including IL-6 and TNF-α) [41–43], acute-phase proteins [42–44], or white blood cell counts [45]. In MDS, elevated levels of IL-6 and IL-1 receptor antagonist and TNF‐α were associated with worse HRQoL [46]. Given the association between inflammation, anemia, and HRQoL, targeting systemic inflammation may be an encouraging therapeutic possibility [10, 47]. For example, treatment with lenalidomide, which modulates different components of the immune system including altering cytokine production [48], has been demonstrated to be associated with improved HRQoL [49–51]. However, it should be noted that improved HRQoL was associated with an increase in hemoglobin level. Other treatment modalities targeting the immune system are currently under study [52]. Furthermore, it has been demonstrated in older patients with hematological malignancies that individuals with malnutrition had more often inflammation (as measured by elevated CRP and low albumin) and suffered from impairments in mood, performance status, and fatigue [18]. Alternatively, the anemia-independent impaired HRQoL in MDS patients could partly be explained by the knowledge of having a (pre-)malignant disorder [36, 53, 54], which is supported by the impact of LR-MDS on the HRQoL dimension anxiety/depression, irrespective of anemia status.

The major strength of this first study assessing the anemia-independent impact of LR-MDS on HRQoL is the use of data from a large MDS registry and a large general population-based cohort. We were able to link data of two well-characterized study cohorts. Another strength is the explicit reporting of our causal assumptions via a causal diagram [33]. This helps to better understand the different components of causal pathways and potential biases. In addition, the huge confounding bias by age, sex, comorbidity, and prior RBC transfusions encoded in the causal diagram is made explicit in Fig. 3 by contrasting the crude (model 1) and confounder-adjusted (model 2) impact estimates. This causal logic supports the plausibility of our results.

Several limitations should be acknowledged. First, HRQoL was assessed using two different general HRQoL questionnaires. By the absence of a cohort including both individuals with MDS and individuals from the general population in which HRQoL was assessed using the same instrument and of whom medical data are available, we used published mapping algorithms to convert the Lifelines data into a summary EQ-5D index score and into scores for the five EQ-5D dimensions. As these mapping algorithms are not perfect, this may have led to measurement bias. On the other hand, the performance of the mapping algorithms is best in the range of EQ-5D index scores relevant for the Lifelines cohort. Absolute estimation errors in the model dataset of the mapping algorithms are smaller than the differences between the two cohorts in this study. Using these algorithms allowed not only the comparison of the overall HRQoL represented by the EQ-5D index but also comparison of mobility, self-care, usual activities, pain/discomfort, and anxiety/depression. A second limitation is that the assessment of comorbidities differed between the cohorts. In the EUMDS cohort, comorbidity was scored according to a physician’s evaluation, whereas in the Lifelines cohort, MDS-CI was mainly based on (verified) medication use and self-reported disorders. Validation studies have shown substantial agreement between self-report questionnaires and medical record data [55–57], but over- or underestimation of the real prevalence cannot be excluded. Third, individuals with a RBC transfusion in the last 30 days were excluded to capture the unmasked impact of anemia, which may have led to selection bias. Fourth, since biochemical measurements associated with inflammation (e.g., CRP or ferritin) or with the type of anemia were only available in a minority of the participants, we were not able to study their relation with HRQoL in the context of MDS. Fifth, as in all observational studies, residual confounding by unknown or unmeasured confounders or the presence of time-varying confounding may bias results [58]. Sixth, there may be additional confounders for the relation between anemia and HRQoL beyond the ones we have adjusted for, which may complicate the “direct” impact estimation. Seventh, there is an ongoing debate on the interpretation of total and “direct” causal impacts of disease characteristics, as changing those needs well-defined interventions. Therefore, our results should be interpreted with caution, merely indicating potential causal pathways that should not be overlooked when optimizing treatment of MDS. Finally, our study is restricted to LR-MDS and cannot be generalized to all subtypes of MDS.

In conclusion, this study presents the first large-scale assessment of the anemia-independent impact of LR-MDS on HRQoL. Our data indicate that the major part of the negative impact of LR-MDS on HRQoL, particularly in the dimensions mobility, usual activities, self-care, and anxiety/depression, is not mediated via anemia. Thus, the therapeutic focus should include treatment strategies directed at underlying pathogenic mechanisms to improve HRQoL, rather than aiming predominantly at increasing hemoglobin levels.

## Supplementary Information

Below is the link to the electronic supplementary material.

Supplementary file1 (DOCX 60 kb)

## Data Availability

Lifelines is a facility which allows data access for reproducibility of the study results. Questions regarding the data availability can be addressed to the Research Office of Lifelines, data@research@lifelines.nl.
