# Supplementary material for: The anemia-independent impact of myelodysplastic syndromes on health-related quality of life
Source: Ann Hematol. 2021 Sep 2;100(12):2921–32. doi: 10.1007/s00277-021-04654-1 (PMC8592948; doi:10.1007/s00277-021-04654-1)
Supplement: Supplementary file 1 — Supplementary file1 (DOCX 60 kb) [file 277_2021_4654_MOESM1_ESM.docx]

**Supplementary Information**

**The anemia-independent impact of myelodysplastic syndromes on health-related quality of life
Annals of Hematology**Hanneke J.C.M. Wouters^1,2^, Annette Conrads-Frank^3^, Karin A. Koinig^4^, Alex Smith^5^, Ge Yu^5,6^, Theo de Witte^7^, Bruce H.R. Wolffenbuttel^2^, Gerwin Huls^1^, Uwe Siebert^3,8-10^, Reinhard Stauder^4^, and Melanie M. van der Klauw^2^, on behalf of the MDS-RIGHT partners ^1^ Dept. of Hematology, University of Groningen, University Medical Center Groningen, Groningen, the Netherlands.
^2^ Dept. of Endocrinology, University of Groningen, University Medical Center Groningen, Groningen, the Netherlands.
^3^ Institute of Public Health, Medical Decision Making and Health Technology Assessment, Dept. of Public Health, Health Services Research and Health Technology Assessment, UMIT - University for Health Sciences, Medical Informatics and Technology, Hall iT, Austria.
^4^ Dept. of Internal Medicine V (Hematology and Oncology), Medical University Innsbruck, Innsbruck, Austria.
^5^ Epidemiology and Cancer Statistics Group, Dept. of Health Sciences, University of York, York, United Kingdom.
^6^ Population Health Sciences Institute, Newcastle University, Newcastle upon Tyne, United Kingdom.
^7^ Dept. of Tumor Immunology, Radboud university medical center, Nijmegen, The Netherlands.
^8^ Division of Health Technology Assessment, ONCOTYROL - Center for Personalized Cancer Medicine, Innsbruck, Austria.
^9^ Center for Health Decision Science, Department of Health Policy and Management, Harvard T.H. Chan School of Public Health, Boston, MA, USA.
^10^ Institute for Technology Assessment and Department of Radiology and Department of Radiology, Massachusetts General Hospital, Harvard Medical School, Boston, MA, USA.

**Corresponding author**Hanneke J.C.M. Wouters, M.D.
Department of Hematology/Endocrinology
University Medical Center Groningen
Hanzeplein 1
P.O. Box 30.001, 9700 RB Groningen, the Netherlands
Phone: 0031 050 361 61 61, Email: h.j.c.m.wouters@umcg.nl

**STable 1. Definitions of comorbidities used for generating MDS-CI in both EUMDS and Lifelines.**

| **Comorbidity** | **Definitions of comorbidities included in the MDS-CI** | **Definitions of comorbidities included in the EUMDS CRF** | **Definitions of comorbidities included in the Lifelines cohort** | **Score if present** |
| --- | --- | --- | --- | --- |
| **Cardiac disease** | - Coronary artery disease (one or more vessel-coronary artery stenosis requiring medical treatment, stent, or bypass graft) - Congestive heart failure - Myocardial infarction - Ejection fraction ≤ 50% | - Coronary artery disease (one or more vessel-coronary artery stenosis requiring medical treatment, stent, or bypass graft)  - Angina  - Previous myocardial infarction  - Heart failure requiring drug treatment  - Ejection fraction ≤ 50% | - Self-reported heart failure - Self reported heart attack with use of platelet aggregation inhibitors (ATC code B01AC) - Self-reported angina - Self-reported balloon angioplasty (stretching of artery with balloon and/or bypass surgery) | 2 |
| **Hepatic disease** | - Liver cirrhosis - Bilirubin > 1.5 × ULN - AST/ALT > 2.5 × ULN | - Liver cirrhosis - Bilirubin > 1.5 x ULN - AST/ALT > 2.5 x ULN | - Self-reported liver cirrhosis  - ALT > 2.5 x ULN - AST > 2.5 x ULN | 1 |
| **Pulmonary disease** | - Diffusion capacity of carbon monoxide and/or FEV1 ≤ 65% - Dyspnea at rest  - Requiring oxygen | - Dyspnea at rest - Requiring oxygen | - Self-reported shortness of breath at rest - FEV1 ≤ 65% | 1 |
| **Renal disease** | - Serum creatinine > 2 mg/dL - On dialysis - Prior renal transplantation | - Serum creatinine > 2 mg/dL - On dialysis | - Serum creatinine > 2 mg/dL - Self-reported prior renal transplantation | 1 |
| **Solid tumour** | Treated at any time point in the patient's past history, excluding nonmelanoma skin cancer | Malignant disease | - Self-reported cancer excluding nonmelanoma skin cancer | 1 |

The final scores are low (0 points), intermediate (1 or 2 points), or high (3 or more points). ALT, alanine aminotransferase; AST, aspartate aminotransferase; ATC, anatomical therapeutic chemical; CRF, case report form; FEV_1_, forced expiratory volume in 1 second; MDS-CI, myelodysplastic syndromes comorbidity index; ULN, upper limit of normal.

**STable 2. EQ-5D index score and percentage of individuals with moderate to severe problems on the five EQ-5D dimensions.**

| **EQ-5D index score and EQ-5D dimensions** | | | | | | | | | | | | | | |
| --- | --- | --- | --- | --- | --- | --- | --- | --- | --- | --- | --- | --- | --- | --- |
|  | **Index score** | | | | **Mobility (%)** | | | | **Self-care (%)** | | | | | |
|  | EUMDS | P-value | Lifelines | P-value | EUMDS | P-value | Lifelines | P-value | EUMDS | | P-value | | Lifelines | P-value |
| **Total** | 0.74 |  | 0.90 |  | 41.1 |  | 13.4 |  | 12.9 | |  | | 1.3 |  |
| **Age group** 50 – 60 years 61 – 75 years  >75 years | 0.78 0.78 0.70 | <0.001 | 0.91 0.90 0.87 | <0.001 | 24.5 33.4 52.1 | <0.001 | 12.5 14.1 22.4 | <0.001 | 4.9 9.0 18.3 | | 0.002 | | 1.2 1.4 2.3 | <0.001 |
| **Sex** Male Female | 0.77 0.69 | <0.001 | 0.92 0.89 | <0.001 | 39.1 44.5 | 0.039 | 10.5 15.6 | <0.001 | 11.5 15.3 | | <0.001 | | 1.0 1.6 | 0.030 |
| **Anemia** Yes No | 0.73 0.83 | <0.001 | 0.88 0.91 | <0.001 | 43.7 19.3 | <0.001 | 18.7 13.2 | <0.001 | 13.8 5.4 | | 0.003 | | 2.2 1.3 | 0.002 |
| **MDS-CI** Low Intermediate High | 0.78 0.72 0.62 | <0.001 | 0.91 0.88 0.83 | <0.001 | 32.8 50.1 71.0 | <0.001 | 11.9 20.5 26.6 | <0.001 | 9.2 16.5 22.6 | | <0.001 | | 1.1 2.0 5.1 | <0.001 |
|  | **Usual activities (%)** | | | | **Pain/discomfort (%)** | | | | **Anxiety/depression (%)** | | | | | |
|  | EUMDS | P-value | Lifelines | P-value | EUMDS | P-value | Lifelines | P-value | EUMDS | P-value | | Lifelines | | P-value |
| **Total** | 34.9 |  | 11.4 |  | 50.3 |  | 44.4 |  | 37.2 |  | | 21.9 | |  |
| **Age group** 50 – 60 years 61 – 75 years  >75 years | 28.7 29.3 41.7 | <0.001 | 11.7 11.4 19.2 | <0.001 | 38.5 44.9 58.2 | <0.001 | 44.0 44.5 51.0 | <0.001 | 44.1 36.4 36.5 | 0.204 | | 23.1 20.1 23.2 | | <0.001 |
| **Sex** Male Female | 32.1 39.5 | 0.003 | 9.1 13.8 | <0.001 | 45.5 58.5 | <0.001 | 38.9 48.7 | <0.001 | 29.9 49.6 | <0.001 | | 17.8 25.0 | | <0.001 |
| **Anemia** Yes No | 36.8 18.7 | <0.001 | 17.2 11.6 | <0.001 | 51.7 38.6 | 0.001 | 47.6 44.3 | 0.018 | 38.2 28.9 | 0.020 | | 24.7 21.8 | | 0.015 |
| **MDS-CI** Low Intermediate High | 30.8 39.6 53.2 | <0.001 | 10.5 17.0 28.1 | <0.001 | 42.4 57.2 69.4 | <0.001 | 43.1 52.1 59.5 | <0.001 | 36.3 34.9 43.5 | 0.422 | | 21.6 27.3 33.6 | | <0.001 |

MDS-CI, myelodysplastic syndromes comorbidity index

**STable 3. The total and anemia-independent ‘direct’ impact of lower-risk myelodysplastic syndromes and the impact of anemia on HRQoL (EQ-5D dimensions scores) based on univariable and multivariable logistic regression analyses.**

| **Mobility** | | | | | | | | |
| --- | --- | --- | --- | --- | --- | --- | --- | --- |
|  | **Model 1 (crude association)** | | **Model 2 (total causal impact)** | | **Model 3 (‘direct’ causal impact)** | | **Model 4 (‘direct’ causal impact)** | |
|  | OR (95% CI) | *P* value | OR (95% CI) | *P* value | OR (95% CI) | *P* value | OR (95% CI) | *P* value |
| **MDS (y/n)** | 4.2 (3.7-4.8) | <0.001 | 2.8 (2.3-3.3) | <0.001 | 2.2 (1.8-2.8) | <0.001 | 2.2 (1.7-2.8) | <0.001 |
| **Anemia (y/n)** |  |  |  |  | 1.3 (1.1-1.6) | 0.008 | 1.3 (1.0-1.6) | 0.032 |
| **Hemoglobin level (g/dL)** |  |  |  |  |  |  | 1.0 (0.9-1.0) | 0.79 |
| **Age group** 50 – 60 years 61 – 75 years  >75 years |  |  | Ref 1.1 (1.0-1.2) 1.9 (1.6-2.3) | 0.19 <0.001 | Ref 1.1 (1.0-1.2) 1.8 (1.5-2.2) | 0.19 <0.001 | Ref 1.1 (1.0-1.2) 1.8 (1.5-2.2) | 0.19 <0.001 |
| **Sex (female)** |  |  | 1.6 (1.5-1.8) | <0.001 | 1.6 (1.5-1.8) | <0.001 | 1.6 (1.5-1.8) | <0.001 |
| **MDS-CI** Low Intermediate High |  |  | Ref 1.9 (1.7-2.1) 2.9 (2.2-3.7) | <0.001 <0.001 | Ref 1.9 (1.7-2.1) 2.9 (2.2-3.7) | <0.001 <0.001 | Ref 1.9 (1.7-2.1) 2.9 (2.2-3.7) | <0.001 <0.001 |
| **RBC transfusion (y/n)** |  |  | 2.1 (1.6-2.9) | <0.001 | 2.1 (1.5-2.8) | <0.001 | 2.1 (1.5-2.8) | <0.001 |
| **Self-care** | | | | | | | | |
|  | **Model 1 (crude association)** | | **Model 2 (total causal impact)** | | **Model 3 (‘direct’ causal impact)** | | **Model 4 (‘direct’ causal impact)** | |
|  | OR (95% CI) | *P* value | OR (95% CI) | *P* value | OR (95% CI) | *P* value | OR (95% CI) | *P* value |
| **MDS (y/n)** | 10.5 (8.4-13.1) | <0.001 | 5.6 (4.0-7.8) | <0.001 | 5.1 (3.0-8.6) | <0.001 | 4.5 (2.6-7.8) | <0.001 |
| **Anemia (y/n)** |  |  |  |  | 1.1 (0.7-1.9) | 0.64 | 0.8 (0.5-1.5) | 0.53 |
| **Hemoglobin level (g/dL)** |  |  |  |  |  |  | 0.9 (0.8-1.0) | 0.041 |
| **Age group** 50 – 60 years 61 – 75 years  >75 years |  |  | Ref 1.0 (0.8-1.3) 2.1 (1.4-3.1) | 0.98 <0.001 | Ref 1.0 (0.8-1.3) 2.1 (1.4-3.0) | 0.98 <0.001 | Ref 1.0 (0.8-1.3) 2.1 (1.4-3.1) | 0.98 <0.001 |
| **Sex (female)** |  |  | 1.5 (1.2-1.9) | <0.001 | 1.5 (1.2-1.9) | <0.001 | 1.4 (1.1-1.8) | 0.007 |
| **MDS-CI** Low Intermediate High |  |  | Ref 1.9 (1.5-2.4) 3.6 (2.3-5.6) | <0.001 <0.001 | Ref 1.9 (1.5-2.4) 3.6 (2.3-5.6) | <0.001 <0.001 | Ref 1.9 (1.5-2.5) 3.5 (2.2-5.4) | <0.001 <0.001 |
| **RBC transfusion (y/n)** |  |  | 2.4 (1.7-3.6) | <0.001 | 2.4 (1.6-3.5) | <0.001 | 2.0 (1.3-3.1) | 0.001 |
| **Usual activities** | | | | | | | | |
|  | **Model 1 (crude association)** | | **Model 2 (total causal impact)** | | **Model 3 (‘direct’ causal impact)** | | **Model 4 (‘direct’ causal impact)** | |
|  | OR (95% CI) | *P* value | OR (95% CI) | *P* value | OR (95% CI) | *P* value | OR (95% CI) | *P* value |
| **MDS (y/n)** | 3.9 (3.4-4.4) | <0.001 | 3.0 (2.5-3.5) | <0.001 | 2.2 (1.8-2.9) | <0.001 | 2.0 (1.6-2.6) | <0.001 |
| **Anemia (y/n)** |  |  |  |  | 1.4 (1.1-1.7) | 0.002 | 1.1 (0.9-1.5) | 0.27 |
| **Hemoglobin level (g/dL)** |  |  |  |  |  |  | 0.9 (0.9-1.0) | 0.003 |
| **Age group** 50 – 60 years 61 – 75 years  >75 years |  |  | Ref 0.9 (0.8-1.0) 1.4 (1.1-1.7) | 0.006 0.001 | Ref 0.9 (0.8-1.0) 1.4 (1.1-1.7) | 0.006 0.002 | Ref 0.9 (0.8-1.0) 1.4 (1.1-1.7) | 0.006 0.002 |
| **Sex (female)** |  |  | 1.6 (1.5-1.8) | <0.001 | 1.6 (1.5-1.8) | <0.001 | 1.5 (1.3-1.7) | <0.001 |
| **MDS-CI** Low Intermediate High |  |  | Ref 1.7 (1.6-1.9) 3.2 (2.5-4.1) | <0.001 <0.001 | Ref 1.7 (1.6-1.9) 3.2 (2.5-4.1) | <0.001 <0.001 | Ref 1.7 (1.6-1.9) 3.2 (2.5-4.1) | <0.001 <0.001 |
| **RBC transfusion (y/n)** |  |  | 2.0 (1.5-2.7) | <0.001 | 2.0 (1.5-2.6) | <0.001 | 1.7 (1.3-2.4) | <0.001 |
| **Pain/discomfort** | | | | | | | | |
|  | **Model 1 (crude association)** | | **Model 2 (total causal impact)** | | **Model 3 (‘direct’ causal impact)** | | **Model 4 (‘direct’ causal impact)** | |
|  | OR (95% CI) | *P* value | OR (95% CI) | *P* value | OR (95% CI) | *P* value | OR (95% CI) | *P* value |
| **MDS (y/n)** | 1.2 (1.0-1.3) | 0.018 | 1.0 (0.8-1.1) | 0.68 | 0.9 (0.8-1.2) | 0.56 | 0.9 (0.8-1.1) | 0.48 |
| **Anemia (y/n)** |  |  |  |  | 1.0 (0.9-1.2) | 0.69 | 1.0 (0.8-1.2) | 0.98 |
| **Hemoglobin level (g/dL)** |  |  |  |  |  |  | 1.0 (1.0-1.0) | 0.51 |
| **Age group** 50 – 60 years 61 – 75 years  >75 years |  |  | Ref 1.0 (0.9-1.0) 1.4 (1.2-1.6) | 0.16 <0.001 | Ref 1.0 (0.9-1.0) 1.4 (1.2-1.6) | 0.16 <0.001 | Ref 1.0 (0.9-1.0) 1.4 (1.2-1.6) | 0.16 <0.001 |
| **Sex (female)** |  |  | 1.5 (1.4-1.6) | <0.001 | 1.5 (1.4-1.6) | <0.001 | 1.5 (1.4-1.6) | <0.001 |
| **MDS-CI** Low Intermediate High |  |  | Ref 1.5 (1.4-1.6) 2.1 (1.6-2.6) | <0.001 <0.001 | Ref 1.5 (1.4-1.6) 2.1 (1.6-2.6) | <0.001 <0.001 | Ref 1.5 (1.4-1.6) 2.1 (1.6-2.6) | <0.001 <0.001 |
| **RBC transfusion (y/n)** |  |  | 1.4 (1.0-1.9) | 0.023 | 1.4 (1.0-1.9) | 0.025 | 1.4 (1.0-1.8) | 0.038 |
| **Anxiety/depression** | | | | | | | | |
|  | **Model 1 (crude association)** | | **Model 2 (total causal impact)** | | **Model 3 (‘direct’ causal impact)** | | **Model 4 (‘direct’ causal impact)** | |
|  | OR (95% CI) | *P* value | OR (95% CI) | *P* value | OR (95% CI) | *P* value | OR (95% CI) | *P* value |
| **MDS (y/n)** | 1.9 (1.7-2.2) | <0.001 | 2.2 (1.9-2.6) | <0.001 | 2.3 (1.8-2.8) | <0.001 | 2.3 (1.8-2.9) | <0.001 |
| **Anemia (y/n)** |  |  |  |  | 1.0 (0.8-1.2) | 0.70 | 1.0 (0.8-1.2) | 0.99 |
| **Hemoglobin level (g/dL)** |  |  |  |  |  |  | 1.0 (1.0-1.1) | 0.51 |
| **Age group** 50 – 60 years 61 – 75 years  >75 years |  |  | Ref 0.8 (0.7-0.9) 0.8 (0.6-0.9) | <0.001 0.007 | Ref 0.8 (0.7-0.9) 0.8 (0.6-0.9) | <0.001 0.007 | Ref 0.8 (0.7-0.9) 0.8 (0.6-0.9) | <0.001 0.007 |
| **Sex (female)** |  |  | 1.6 (1.5-1.8) | <0.001 | 1.6 (1.5-1.8) | <0.001 | 1.7 (1.5-1.8) | <0.001 |
| **MDS-CI** Low Intermediate High |  |  | Ref 1.4 (1.3-1.5) 1.9 (1.5-2.4) | <0.001 <0.001 | Ref 1.4 (1.3-1.5) 1.9 (1.5-2.4) | <0.001 <0.001 | Ref 1.4 (1.3-1.5) 1.9 (1.5-2.4) | <0.001 <0.001 |
| **RBC transfusion (y/n)** |  |  | 1.2 (0.9-1.6) | 0.19 | 1.2 (0.9-1.6) | 0.18 | 1.2 (0.9-1.7) | 0.15 |

Model 1 was a crude analysis. Model 2 was adjusted for age (50-60 years, 61-75 years or 75 years and older), sex, comorbidity (MDS-CI low, intermediate, high) and prior RBC transfusions. Model 3 was additionally adjusted for anemia. Model 4 was additionally adjusted for hemoglobin levels. CI, confidence interval; MDS, myelodysplastic syndromes; MDS-CI, myelodysplastic syndromes comorbidity index; OR, odds ratio; RBC, red blood cell; Ref, reference.

**STable 4. Sensitivity analysis including in EUMDS only individuals with IPSS-R (very) low risk who never received a RBC transfusion for the total and anemia-independent ‘direct’ impact of lower-risk myelodysplastic syndromes on HRQoL and the impact of anemia on HRQoL (EQ-5D index score and dimensions scores).**

|  | **EQ-5D index score** | | | | **Mobility** | | **Self-care** | |
| --- | --- | --- | --- | --- | --- | --- | --- | --- |
|  | **Model 4 (‘direct’ causal impact)** | | | | **Model 4 (‘direct’ causal impact)** | | **Model 4 (‘direct’ causal impact)** | |
|  | B | SE | β | *P* value | OR (95% CI) | *P* value | OR (95% CI) | *P* value |
| **MDS (y/n)** | -0.105 | 0.004 | -0.141 | <0.001 | 2.3 (1.8-3.0) | <0.001 | 4.5 (2.5-8.0) | <0.001 |
| **Anemia (y/n)** | -0.022 | 0.003 | -0.044 | <0.001 | 1.3 (1.0-1.7) | 0.034 | 0.9 (0.5-1.7) | 0.91 |
| **Hemoglobin level (g/dL)** | 0.001 | 0.001 | 0.006 | 0.35 | 1.0 (1.0-1.1) | 0.94 | 0.9 (0.8-1.0) | 0.056 |
| **Age group** 50 – 60 years 61 – 75 years  >75 years | Ref -0.001 -0.036 | 0.001 0.003 | -0.004 -0.061 | 0.36 <0.001 | 1.1 (1.0-1.2) 1.8 (1.4-2.2) | Ref 0.19 <0.001 | Ref 1.0 (0.7-1.3) 1.7 (1.1-2.6) | 0.96 0.026 |
| **Sex (female)** | -0.023 | 0.001 | -0.108 | <0.001 | 1.6 (1.4-1.8) | <0.001 | 1.43(1.0-1.7) | 0.10 |
| **MDS-CI** Low Intermediate High | Ref -0.032 -0.080 | 0.002 0.006 | -0.078 -0.062 | <0.001 <0.001 | Ref 1.9 (1.7-2.1) 2.9 (2.2-3.7) | <0.001 <0.001 | Ref 2.1 (1.6-2.7) 3.7 (2.2-6.1) | <0.001 <0.001 |
|  | **Usual activities** | | | | **Pain/discomfort** | | **Anxiety/depression** | |
|  | **Model 4 (‘direct’ causal impact)** | | | | **Model 4 (‘direct’ causal impact)** | | **Model 4 (‘direct’ causal impact)** | |
|  | OR (95% CI) | | *P* value | | OR (95% CI) | *P* value | OR (95% CI) | *P* value |
| **MDS (y/n)** | 1.9 (1.5-2.5) | | <0.001 | | 0.9 (0.7-1.1) | 0.29 | 2.4 (1.9-3.0) | <0.001 |
| **Anemia (y/n)** | 1.1 (0.9-1.5) | | 0.35 | | 1.0 (0.8-1.2) | 0.97 | 1.0 (0.8-1.2) | 0.94 |
| **Hemoglobin level (g/dL)** | 0.9 (0.9-1.0) | | 0.004 | | 1.0 (1.0-1.0) | 0.43 | 1.0 (1.0-1.1) | 0.33 |
| **Age group** 50 – 60 years 61 – 75 years  >75 years | Ref 0.9 (0.8-1.0) 1.4 (1.1-1.7) | | 0.005 0.004 | | Ref 1.0 (0.9-1.0) 1.3 (1.1-1.5) | 0.15 0.010 | Ref 0.8 (0.7-0.9) 0.8 (0.6-1.0) | <0.001 0.015 |
| **Sex (female)** | 1.4 (1.3-1.6) | | <0.001 | | 1.5 (1.4-1.6) | <0.001 | 1.7 (1.5-1.8) | <0.001 |
| **MDS-CI** Low Intermediate High | Ref 1.8 (1.6-2.0) 3.5 (2.7-4.5) | | <0.001 <0.001 | | Ref 1.5 (1.4-1.6) 2.0 (1.6-2.5) | <0.001 <0.001 | Ref 1.4 (1.3-1.6) 1.8 (1.4-2.3) | <0.001 <0.001 |

Model 4 was adjusted for age (50-60 years, 61-75 years or 75 years and older), sex, comorbidity (MDS-CI low, intermediate, high), anemia and hemoglobin levels. B, unstandardized regression coefficient; β, standardized regression coefficient; CI, confidence interval; MDS, myelodysplastic syndromes; MDS-CI, myelodysplastic syndromes comorbidity index; Ref, reference; OR, odds ratio; SE, standard error.

**STable 5. List of EUMDS participants contributing to this study.**

| **Country** | **Participating centers** (City, Name organisation, *[local investigator(s)]*) |
| --- | --- |
| **Austria** | Innsbruck, Medical University of Innsbruck *[****R. Stauder****]*  Wels, Klinikum Kreuzschwestern *[S. Burgstaller / J. Thaler]*  Lienz, Bezirkskrankenhaus *[A. Walder]*  Vienna, Hanusch Krankenhaus *[M. Pfeilstöcker / A. Schoenmetzler-Makrai]* |
|  |  |
|  |  |
| **Croatia** | Zagreb, Clinical Hospital Merkur *[****I. Mandac Rogulj****]* |
| **Czech**  **Republic** | Prague, General University Hospital, Institute of Hematology and Blood Transfusion  *[****J. Cermak*** */ D. Mikulenková]*  Prague, University Hospital Motol *[I. Hochova]*  Brno, The University Hospital Brno *[M. Krejci]*  Prague, General University Hospital, 1st Clinic of Internal Medicine *[A. Jonasova]*  Hradec Kralove, Charles University Faculty of Medicine *[J. Voglova]*  Olomouc, University Hospital *[P. Rohon]* |
|  |  |
| **Denmark** | Aarhus, University Hospital *[****MS. Holm****]*  Odense, Odense University Hospital *[H. Vestergaard]*  Copenhagen, University Hospital: Rigshospitalet *[L. Kjeldsen]*  Herlev Ringvej, Herlev Hospital *[IH. Dufva]*  Aalborg, University Hospital *[PD. Jensen]* |
|  |  |
|  |  |
| **France** | Bobigny, Hospital Avicenne *[****P. Fenaux****]*  Paris, Hôpital St Louis *[****P. Fenaux*** */ R. Itzykson / L. Adès]*  Nancy, CHU Nancy: Hospital Brabois (Vandoeuvre Les Nancy) *[A. Guerci]*  Avignon, Centre Hospital *[B. Slama]*  Perpignan, Centre Hospital Maréchal Joffre *[L. Sanhes]*  Toulouse, CHU Toulouse: Hospital Purpan, Toulouse *[O. Beyne-Rauzy]*  Lyon, Hospital Edouard Herriot *[E. Wattel]*  Cochin, Hospital Hôtel Dieu *[L. Willems]*  Chalon sur Saone, Centre Hospital William Morey *[D. Klepping / B. Salles]*  Le Kremlin Bicêtre, Hospital Bicêtre *[G. Tertian]*  Limoges, CHU Limoges Hospital Dupuytren *[M. Chaury]*  Tours, CHRU de Tours *[E. Gyan]*  Nice, CHU de Nice: Hospital l'Archet *[L. Legros]*  Strasbourg, CHU Hospital Hautepierre de Strasbourg *[S. Amé]*  Caen, Centre Hospital Universitaire Clemenceau *[S. Cheze]*  Antibes, Hospital Center D'antibes Juan-Les-Pins *[D. Re]*  Boulogne sur Mer, Centre Hospital Boulogne-sur-Mer *[B. Choufi]*  Rouen, CHU de Rouen: Hospital Charles-Nicolle *[A. Stamatoullas]*  Fréjus, CHI Frejus Saint Raphael *[J. Gutnecht]*  Grenoble, CHU Albert Michallon *[S. Courby]*  Lille - St Vincent, Hospital St Vincent de Paul *[L. Pascal]*  Pontoise, Centre Hospital de Pontoise *[R. Benramdane ]* |
| **Germany** | Dresden, University Hospital Carl Gustav Carus *[U. Platzbecker]*  Düsseldorf, Heinrich-Heine University Hospital *[****U. Germing****]*  Duisburg, HELIOS: St. Johannes Hospital in Hamborn *[C. Badrakan]*  Freiburg, University Hospital Freiburg *[M. Lübbert]*  Ulm, University Hospital Ulm *[R. Schlenk]* |
|  |  |
| **Greece** | Patras, General University Hospital of Patras *[****A. Symeonidis*** */ A. Kourakli]*  Athens, General Hospital Laikon - Propaedeutic Medicine, University of Athens Medical School *[P. Panagiotidis]*  Athens, Patission Prefectural General Hospital: Halkida *[Z. Kartasis]*  Athens, Pammakaristos Hospital *[A. Kostourou]*  Alexandroupolis, Democritus University of Thrace *[I. Kotsianidis / C. Tsatalas]*  Piraeus, Metaxa Hospital *[M. Kotsopoulou / K. Megalakaki]*  Chania, General Hospital of Chania *[K. Palla]*  Thessaloniki, Hippokration - General Hospital of Thessaloniki *[E. Vlachaki]*  Athens, General Hospital Laikon - Internal Medicine, University of Athens Medical School *[N. Viniou]*  Ioannina, University Hospital of Ioannina *[V. Briasoulis / E. Hatzimichael]*  Athens, General Hospital Attikon, University of Athens Medical School *[V. Pappa]*  Serres, General Hospital of Serres *[M. Protopapa]*  Athens, General Hospital G.Gennimatas *[A. Galanopoulos / E. Michali]*  Athens, General Hospital Sotiria, University of Athens Medical School *[A. Katsigiannis / P. Roussou]*  Pilea Chortiatis, General Hospital of Thessaloniki George Papanikolaou *[A. Anagnostopoulos]*  Thessaloniki, Theageneio General Hospital *[P. Konstantinidou]* |
| **Israel** | Tel Aviv, Tel Aviv Sourasky Medical Centre *[****M. Mittelman****]*  Beersheba, Soroka Medical Center *[E. Levy / U. Greenbaum]*  Rehovot, Kaplan Medical Center *[K. Filanovsky]*  Kfar Saba, Meir Medical Center *[I. Hellman / M. Ellis]*  Haifa, Rambam Medical Centre *[Y. Ofran]*  Nahariya, The Western Galilee Hospital *[A. Braester]*  Afula, HaEmek Medical Center *[G. Stemer]*  Ashkelon, Barzilai Medical Center *[A. Nemetz]*  Be'er Ya'akov, Asaf-Harofe Medical Center *[U. Gotwin / O. Cohen / M. Koren]*  Petah Tikva, Rabin Medical Center *[G. Itzhaki / O. Wolaj]*  Haifa, Carmel Medical Center *[M. Price]*  Haifa, Bnai Zion Medical Center *[S. Gino-Moor]*  Jerusalem, Hadassah Medical Center *[N. Goldshmidt / S. Elias / R. Sabag]*  Tiberias, Baruch Padeh Medical Center Poriya *[S. Yeganeh / O. Katz]* |
|  |  |
| **Italy** | Pavia, IRCCS San Matteo Hospital Foundation *[****L. Malcovati****]*  Rome, University Cattolica del Sacro Cuore, Policlinico Gemelli *[L. Fianchi]* |
|  |  |
| **Netherlands** | Nijmegen, Radboudumc *[****S. Langemeijer*** */ M. MacKenzie]*  Ede, Gelderse Vallei Hospital *[G. Velders]*  Arnhem, Rijnstate Hospital *[V. Matthijssen]*  Den Bosch, Jeroen Bosch Hospital *[A. Herbers / H. Pruijt]*  Uden, Bernhoven Hospital *[C. Lensen]*  Amsterdam, VU University Medical Center *[A. vd Loosdrecht]*  Doetinchem, Slingeland Hospital *[N. Aboosy / F. de Vries]*  Helmond, Elkerliek Hospital *[E. Jacobs]*  Veldhoven, Maxima Medical Center *[P. Kuijper]* |
| **Poland** | Warsaw, Warszawski Uniwersytet Medyczny *[****K. Madry****]* |
| **Romania** | Bucharest, Fundeni Clinical Institute *[****A. Tatic****]*  Bucharest, Coltea Clinical Hospital *[O. Stanca Ciocan]*  Brasov, Districtual Hospital *[G. Vulkan]* |
|  |  |
| **Serbia** | Novi Sad, Clinical Center of Vojvodina *[****A. Savic****]* |
| **Spain** | Oviedo, Hospital Universitario Central de Asturias *[T. Bernal]*  Valencia, Hospital Clinico Universitario de Valencia *[D. Tormo]*  Lleida, Instituto de Investigación Biomédica *[V. Betés]*  Barcelona, Hospital del Mar *[C. Pedro]*  Salamaca, Hospital Universitario de Salamanca *[M. Diez Campelo]*  Valencia, Hospital Dr. Peset *[R. Andreu Lapiedra]*  Valencia, Hospital Universitario La Fe *[****G. Sanz****]*  Barcelona, Hospital Universitari Germans Trias i Pujol *[B. Xicoy]*  Murcia, Hospital Universitario Meseguer *[M. Lozano / M. Martínez]*  Cádiz, Hospital Del Sas, Jerez De La Frontera *[P. Leiva]*  Cádiz, Hospital Universitario Puerta del Mar *[J. Muñoz]*  Murcia, Hospital Universitario Virgen de la Arrixaca *[P. Iniesta]* |
|  |  |
| **Sweden** | Stockholm, Karolinska University hospital *[****E.Hellström-Lindberg****]*  Göteborg, Sahlgrenska University Hospital *[H. Garelius]*  Stockholm, Södersjukhuset *[M. Grövdal]*  Luleå, Sunderby Hospital *[L. Brandefors]*  Umeå, Umeå regional hospital *[F. Lorenz]*  Linköping, University Hospital Linköping *[P. Antunovic / A. Jönsson]*  Örebro, Örebro University Hospital *[P.Kozlowski]*  Uppsala, Uppsala University *[E. Ejerblad]*  Eskilstuna, Mälarsjukhuset *[E. Hesse Sundin]*  Halmstad, Teaching Hospital of Halmstad *[C. Karlsson]* |
| **United**  **Kingdom** | Leeds, Leeds Teaching Hospitals *[****D. Bowen*** */ M. Karakantza]*  Aberdeen, Aberdeen Royal Infirmary *[D. Culligan]* Blackpool, Blackpool Victoria Hospital *[P. Cahalin / S. Kolade]*  Worcester, Worcestershire Acute Hospitals NHS Trust *[J. Mills]*  Northampton, Northampton General Hospital *[J. Parker / A. Bowen]*  Steeton, Airedale NHS Trust *[E. Nga]*  Bradford, Bradford Royal Infirmary *[S. Ackroyd]*  Bournemouth, Royal Bournemouth Hospital *[S. Killick]*  Harrogate, Harrogate District Hospital *[C. Hall]*  Truro, Royal Cornwall Hospital *[D. Creagh]*  York, York Hospital *[L. Bond]*  Wakefield, Mid Yorkshire Hospitals *[J. Ashcroft]*  Cambridge, Addenbrooke's Hospital *[C. Wong / A. Warren]*  Nottingham, City Hospital *[R. Radia / E. Das-Gupta]*  Glasgow, Western Infirmary *[M. Drummond]*  Oxford, John Radcliffe Hospitals NHS Trust *[P. Vyas]*  Huddersfield, Huddersfield Royal Infirmary *[K. Rothwell]*  Newcastle upon Tyne, Royal Victoria Infirmary *[G. Jones]*  Hull, Hull and East Yorkshire Hospitals NHS Trust *[S. Green / S. Ali]*  Manchester, Christie Hospital *[M. Dennis]* |
|  |  |

EUMDS SC members are underlined; **Country PI’s** are bold; sorted per country by descending number of patients within this study.
